# Case of a Fracture of the Scull Successfully Treated

**Published:** 1786

**Authors:** John Causer

**Affiliations:** Member of the Corporation of Surgeons of London, and Surgeon at Stourbridge, in Worcestershire


### VII. *Case of a Fracture of the Scull successfully treated. Communicated in a Letter to Dr.* Simmons *by Mr.* John Causer, *Member of the Corporation of Surgeons of London, and Surgeon at Stourbridge, in Worcestershire.*

AS the prevalent and succefsful practice of healing wounds without aiming at suppuration has lately been extended to fractures of the scull, where the use of the trephine was indispensable; and as this great improvement in surgery has not hitherto been supported by a sufficient number of facts, permit me to addrefs the following case to you, hoping you will give it a place in your valuable Medical Journal, as the extensive circulation of it in that work may probably be the means of saving some lives, and of preventing much trouble and anxiety to my brother practitioners.

### CASE.

Lewis Holmes, an healthy boy, fourteen years old, on the 22d of September, 1785, being kicked on his forehead by a horse, was struck to the ground, and remained senseless about half an hour. About an hour after the accident I saw him, lying in a comatose state, with with a wound on his forehead more than two inches in length, and, on examining it, I found a fracture, with great comminution, as well as confiderable depreffion of that portion of the frontal bone which lies immediately above the middle of the right orbit.

The wound was formed nearly as a fingle line, and paffed obliquely upwards and outwards in that part of the fcalp, under which the temporal mufcle becomes tendinous; fo that by making a fimple incifion with a knife at each extremity I difcovered the extent of the fracture to be full four inches.

I then, beginning at each edge of the wound, carefully diffected the fcalp from the pericranium, which covered the broken fragments of bone, and having extended the feparation of it fufficiently upwards to make room for the application of the trephine, made one perforation in the found bone near to the upper edge of the fracture. I next raifed the depreffed portions of bone with the elevator, and with the forceps removed nineteen fmall broken pieces of different fizes, which occafioned a very confiderable lofs of boney fubftance, and left the edges of the found bone with many irregular points.

Nearly in the central part of the dura mater, expofed to view, I obferved a laceration of that membrane about half an inch in length, which had been made by the fractured pieces of bone: from which circumftance, and apprehending danger of a troublefome fungus arifing, (as had twice happened to me) I was determined to treat the wound in the method practifed by Mr. Mynors, and recommended in his late publication*, in preference to the ufual plan, though the bringing the fcalp over the irregular points of bone appeared to me likely to occafion much irritation and pain, and unpromifing of fuccefs. I therefore cleanfed the dura mater and the reft of the wound with a fponge and warm water; then extended the portions of fcalp, and laying them immediately upon the dura mater, retained their edges in clofe contact by means of two ligatures and fmall ftrips of fticking plafter; over which I applied a folded linen rag fpread with a mild cerate; covering the whole with a handkerchief applied in the ufual manner.

* See his Hiftory of the Practice of trepanning the Scull, and the after Treatment; with Obfervations on a new Method of Cure.

The

The boy was now fenfible, and, being put to bed, took a pill, containing *opii & ipecacoanhæ* āā *gr. j.*; a few drops of a mixture of thebaic tincture and antimonial wine were ordered to be given him every four or five hours.

Sept. 23, he had paffed a good night; his pulfe was regular, and his fkin moift; he had no head-ach or thirft, and complained but of very little pain in the injured part: his bowels were opened by a folution of Glauber's falt, and the drops were continued.

24th, He had a good night, free from fever or pain.

25th, The dreffings were removed, and the fcalp was found to be every where united, except at the fuperior part of the wound, where a very fmall portion of bone was vifible, and from which granulations were rifing. The digeftion was very fmall. A mild cerate was now applied over the edges of the wound in the fcalp fuperficially, fpread on lint, over which I placed ftrips of fticking plafter. The boy made no complaint, except that of being very hungry.

26th, I cut the ligatures, and drew them out; after which I applied the fame dreffings as before; and the boy, being now in good health, health, would no longer be confined either to the houſe or his low diet. I afterwards dreſſed the wound every other day till it was quite healed. The laſt dreſſing was on the 8th of October, the ſixteenth day from the accident. I have repeatedly ſeen the patient ſince the cure has been compleated: he continues perfectly well; no exfoliation has taken place; and the ſcalp is healed ſo ſmooth and even, that there is no deformity of the leaſt conſequence.

The event of this caſe, when we conſider the diſagreeable circumſtances which attended it, viz. the *jagged points of the edge of the bone left in the wound*, and made by the irregularity of the fracture, and *the laceration of the dura mater*, has very fully convinced me that the plan which I adopted is a very improved method of treating ſuch accidents, both for expedition of the cure, and ſafety to the patient, and it is a practice I ſhall ever purſue in future.

*Stourbridge*,
March 2, 1786.

VIII. *Expe-*

